# The role of the tumour microenvironment in the angiogenesis of pituitary tumours

**DOI:** 10.1007/s12020-020-02478-z

**Published:** 2020-09-18

**Authors:** Pedro Marques, Sayka Barry, Eivind Carlsen, David Collier, Amy Ronaldson, Neil Dorward, Joan Grieve, Nigel Mendoza, Ramesh Nair, Samiul Muquit, Ashley B. Grossman, Márta Korbonits

**Affiliations:** 1 Centre for Endocrinology, William Harvey Research Institute, Barts and the London School of Medicine and Dentistry, Queen Mary University of London, London, UK; 2 Department of Pathology, STHF, Skien, Norway; 3 The National Hospital for Neurology and Neurosurgery, UCLH, NHS Trust, London, UK; 4 Department of Neurosurgery, Charing Cross Hospital, Imperial College, London, UK; 5 Department of Neurosurgery, Derriford Hospital, Plymouth, UK

**Keywords:** Pituitary neuroendocrine tumour, Pituitary adenoma, Tumour microenvironment, Cytokine, Immune cell, Angiogenesis

## Abstract

**Purpose:**

Angiogenesis has been studied in pituitary neuroendocrine tumours (PitNETs), but the role of the tumour microenvironment (TME) in regulating PitNET angiogenesis remains unknown. We aimed to characterise the role of TME components in determining the angiogenetic PitNET profile, focusing on immune cells and tumour-derived cytokines.

**Methods:**

Immune cells were studied by immunohistochemistry in 24 human PitNETs (16 non-functioning-PitNETs (NF-PitNETs) and 8 somatotrophinomas): macrophages (CD68, CD163, HLA-DR), cytotoxic (CD8) and T helper (CD4) lymphocytes, regulatory T cells (FOXP3), B cells (CD20) and neutrophils (neutrophil elastase); endothelial cells were assessed with CD31. Five normal pituitaries (NP) were included for comparison. Microvessel density and vascular morphology were estimated with ImageJ. The cytokine secretome from these PitNETs were assessed on culture supernatants using a multiplex immunoassay panel.

**Results:**

Microvessel density/area was higher in NP than PitNETs, which also had rounder and more regular vessels. NF-PitNETs had vessels of increased calibre compared to somatotrophinomas. The M2:M1 macrophage ratio correlated with microvessel area. PitNETs with more CD4+ T cells had higher microvessel area, while tumours with more FOXP3+ cells were associated with lower microvessel density. PitNETs with more B cells had rounder vessels. Of the 42 PitNET-derived cytokines studied, CCL2, CXCL10 and CX3CL1 correlated with microvessel density and vessel architecture parameters.

**Conclusions:**

M2 macrophages appear to play a role in PitNET neovascularisation, while B, CD4+ and FOXP3+ lymphocytes, as well as non-cellular TME elements such as CCL2, CXCL10 and CX3CL1, may also modulate the angiogenesis of PitNETs.

## Introduction

The great majority of pituitary neuroendocrine tumours (PitNETs) are benign, although they can be associated with significant morbidity due to mass effects on surrounding tissues and/or excessive or low hormone secretion [1, 2]. PitNETs may invade the cavernous sinus or other nearby structures, and may be refractory to conventional treatment or recur despite optimal surgical and/or medical therapy [2, 3]. Different mechanisms may contribute to increased PitNET invasiveness, including interactions within the tumour microenvironment (TME) which may modulate several tumourigenic processes such as tumour cell proliferation [4, 5], tumour cell migration and invasion [6–8] and immune cell chemoattraction [9], and this may also include angiogenesis [9, 10].

Angiogenesis is the process by which new blood vessels are formed from pre-existing ones, and is essential for tumour development, growth, invasion and metastasis. Angiogenesis is regulated by different non-cellular TME components, such as cytokines, chemokines, growth factors or extracellular matrix-remodelling enzymes [11–13], as well as by different non-neoplastic cells such as macrophages [14] or tumour-associated fibroblasts [15]. The degree of tumour angiogenesis is commonly evaluated by assessment of microvessel density, i.e. the number of vessels per given area, although other vascular morphological parameters are also relevant [11], and studying angiogenesis often relies on the CD31 and CD34 endothelial cell antigens [16].

Some previous studies have investigated angiogenesis in PitNETs [11–13, 17–23]. However, research specifically studying the role of the different elements of the TME in the modulation of PitNET neovascularisation is scarce [24], contrasting with the extensive data available for other cancers [25–27]. Exploring this further within the context of the complex TME interactions may provide invaluable insights in PitNET pathophysiology and therapeutic advances for aggressive PitNETs, particularly with the employment of anti-angiogenic drugs, such as bevacizumab (an anti-vascular endothelial growth factor (VEGF) monoclonal antibody) [28, 29], or check-point inhibitors [30–32]. In this study, we aimed to characterise the role of various TME elements underlying pituitary tumour angiogenesis, focusing on infiltrating immune cells and the PitNET-derived cytokine network.

## Material and methods

### Human PitNET and normal pituitary samples

Fresh tissues from 8 somatotrophinomas and 16 clinically non-functioning PitNETs (NF-PitNETs) were collected at the time of pituitary surgery; a fragment was used for primary culture, while paraffin-embedded tumour tissue sections were used for immunohistochemical studies. Analysis of certain features of the TME in these samples have been previously published, where further details regarding patient samples can be obtained [9]. Normal pituitary (NP) autopsy samples from five individuals with no endocrine, immune or malignant disease were included for comparison. Clinico-pathological data from each patient were collected from medical records. Blood samples for the measurement of serum pituitary hormones were routinely taken at diagnosis and before the pituitary surgery, and assayed in a certified National Health Service laboratory. This study was approved by the Cambridge East Research Ethics Committee (MREC No. 06/Q0104/133).

### Immunohistochemical analysis

Immunostains were done on paraffin-embedded sections using the Ventana Discovery DAB Map System (Ventana, Illkirch, France), as previously described [9]. We assessed macrophages using CD68 (anti-CD68, DAKO IR613, dilution 1:2), CD163 for M2-like macrophages (anti-CD163, Abcam Ab74604, neat) and HLA-DR for M1-like macrophages (anti-HLA-DR, Abcam Ab20181 [TAL1B5], dilution 1:100) [14, 33–35], lymphocytes using CD8 for cytotoxic T cells (anti-CD8, DAKO M7103, dilution 1:100), CD4 for T helper cells (anti-CD4, Abcam Ab133616, dilution 1:100), FOXP3 for T regulatory cells (anti-FOXP3, Abcam Ab20034 [236 A/E7], dilution 1:50), CD20 for B lymphocytes (anti-CD20, DAKO M0755, dilution 1:300) and neutrophil elastase for neutrophils (anti-neutrophil elastase, Abcam Ab68672, dilution 1:100). To study endothelial cells we used the marker CD31 (anti-CD31, DAKO M0823, dilution 1:100), and we also took into consideration their location and morphology. Stained slides were scanned with the Pannoramic 250 High Throughput Digital Slide Scanner and analysed with the Pannoramic Viewer Software (3DHISTECH, Budapest, Hungary). Full slides were first verified on haematoxylin and eosin stained sections, then inspected at low magnification to identify “hot spot” areas, and then immunopositive cells were counted in 5 high-power field (HPF) as described in [9]. The PitNET-infiltrating immune cell thresholds considered here were the same as those previously published in [9]. Vessels, stained for CD31, were counted in 3 “hot spot” ×20 magnification fields per case to calculate the number of vessels per HPF (microvessel density), and the vessels’ contour was traced manually with ImageJ (National Institutes of Health, USA) to calculate the percentage (%) of the microvessel area per HPF (total microvessel area), as previously reported [10, 11]. Vascular architecture parameters are expressed as follows: perimeter and Feret’s diameter (longest distance between any two points along selection boundary) in µm; area occupied per vessel in percentage (%) of the HPF; roundness (representing vessel shape, 4 × [Area]/π × [Major axis]^2^) is expressed with a numeric value comprised between 0 and 1 (the value 1 corresponding to a perfect circle shape and 0 for very elongated vessels).

### Primary culture

Fresh PitNET tissue was collected in complete medium and processed as described in [9]. Tryptan blue solution (Sigma, cat. no. T8154) was used to assess viable cells, and if viability >90% 2 million cells were seeded per well in complete medium in a six-well plate coated with poly-L-lysine (Sigma, cat. no. P4707) and incubated at 5% CO_2_ at 37 °C. The supernatants were generated after 24 h on 1 mL serum-free medium, and assayed by Eve Technologies (Calgary, Alberta, Canada) according to their protocol using the human cytokine/chemokine array with IL-18 (HD42) kit (Millipore, St. Charles, USA), as previously described [9]. This array measured 42 different cytokines, chemokines and growth factors in the same sample: G-CSF, GM-CSF, IFNα2, IFNγ, IL-1α, IL-1β, IL-1ra, IL-2, IL-3, IL-4, IL-5, IL-6, IL-7, IL-8, IL-9, IL-10, IL-12(p40), IL-12(p70), IL-13, IL-15, IL-17A, IL-18, CXCL1, CXCL10, CCL2, CCL3, CCL4, CCL5, CCL7, CCL11, CCL22, CX3CL1, sCD40L, Flt-3L, PDGF-AA, PDGF-BB, TGF-α, TNF-α, TNF-β, VEGF-A, EGF and FGF-2.

### Statistical analysis

Statistical analyses were carried out using the SPSS statistical software v20 (IBM, USA) and GraphPad v6 (Prism, USA). Continuous variables were tested for Gaussian distribution with the Shapiro–Wilk test, and the non-parametric quantitative data analysed with the Mann–Whitney *U* test or the Kruskal–Wallis test, as appropriate. Data are shown as median (interquartile range). Correlations between continuous variables (non-normally distributed) were determined by the Spearman’s correlation coefficient rho. *P* values < 0.05 were considered significant.

## Results

### Angiogenesis in human PitNETs versus NPs

**Fig. 1:**
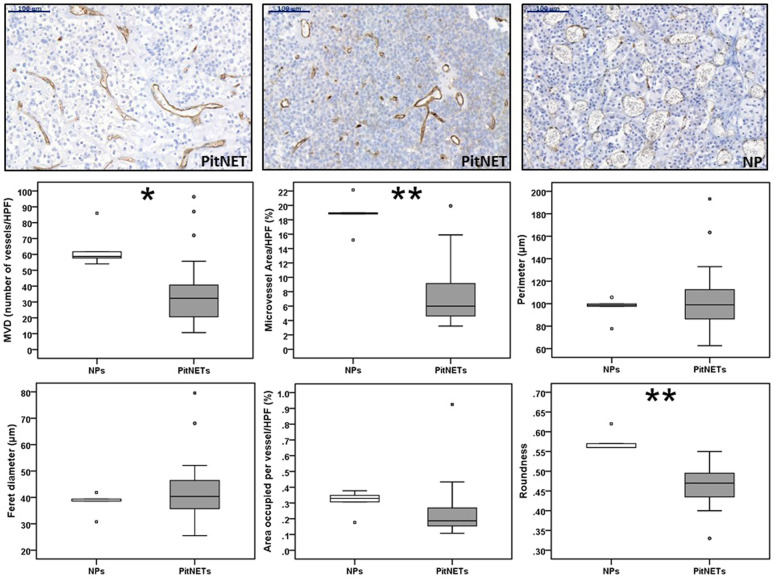
Angiogenesis in human pituitary neuroendocrine tumours and in normal pituitary. Microvessel density (MVD) and vasculature architecture parameters differences between human pituitary neuroendocrine tumours (PitNETs) and normal pituitaries (NPs) are shown. PitNETs (*n* = 24) and NPs (*n* = 5) tissue sections were stained for CD31. CD31 positive vessels were counted in three different high-power fields (HPF) to obtain MVD (number of vessels/HPF). CD31-stained ×20 magnification fields were analysed with ImageJ and vessel contour was manually traced in ImageJ in order to obtain the vasculature architecture parameters: total microvessel area, area occupied per vessel, vessel perimeter, vessel Feret’s diameter and roundness (vessel roundness correspond to a value comprised between 0 and 1, with 1 = perfect circle). Representative images of vessels from two PitNETs and one NP are shown (×20). Scale bar 100 µm. *p < 0.05, **p < 0.01, ***p < 0.001 (Mann–Whitney *U* test)

We analysed a cohort of 24 PitNETs with clinico-pathological, cytokine and infiltrating immune cells data for microvessel density and vasculature architecture parameters, staining the vessels with CD31. The vasculature significantly differed between PitNETs and NPs (Fig. 1). When compared to NP, PitNETs had a remarkably lower microvessel density (32.3 (20.5–40.7) vs 58.7 (55.8–73.8) vessels/HPF; *p* = 0.011) and lower microvessel area (6.0 (4.4–9.3) vs 18.9 (17.0–20.6) % of microvessel area/HPF; *p* = 0.001). In terms of vascular architecture parameters, there were no major differences between PitNETs and NPs, except that vessels were less round in PitNETs than those seen in NP (0.47 (0.43–0.49) vs 0.56 (0.56–0.59); *p* = 0.001) (Fig. 1).

### Clinico-pathological features and angiogenesis in PitNET patients

Of the 24 PitNET patients included in the study, 16 were males (66.7%), 10 had cavernous sinus invasion (41.7%), 5 cases had a Ki-67 ≥ 3% (20.8%), and the patients’ median age at diagnosis was 48.5 (39.3–61.8) years. According to the Trouillas clinico-pathological classification [3], 11 patients were classified as 1a (non-proliferative and non-invasive), 3 patients as 1b (proliferative but non-invasive), while 8 and 2 patients were classified, respectively, as 2a (invasive but non-proliferative) and 2b (invasive and proliferative). Five PitNET samples were obtained from patients who were re-operated (Table 1). Overall, there was no association between microvessel density, total microvessel area or vascular architecture parameters and the different clinico-pathological features among PitNET patients, in particular regarding the presence of cavernous sinus invasion, increased Ki-67, or among the different PitNET grades according to the Trouillas classification [3]. Re-operated PitNETs tended to have an increased microvessel density (*p* = 0.072) and vessel roundness (*p* = 0.074) compared to PitNETs operated for the first time (Table 1).

**Table 1:** Angiogenesis and clinical features in patients with PitNETs

	MVD (number of vessels/HPF)	TMVA (% vessel area/HPF)	Perimeter (µm)	Feret’s diameter (µm)	Area per vessel (% vessel area/HPF)	Roundness (0–1)
Gender						
Male (*n* = 16)	31.7 (20.0–39.7)	6.0 (4.9–8.8)	98.7 (86.1–117.1)	40.1 (35.4–47.2)	0.18 (0.15–0.28)	0.47 (0.43–0.53)
Female (*n* = 8)	35.8 (20.7–64.2)	7.2 (3.9–14.3)	100.9 (83.9–113.1)	41.4 (34.0–46.0)	0.21 (0.14–0.28)	0.47 (0.46–0.48)
Headache at diagnosis						
Yes (*n* = 8)	31.8 (20.7–39.3)	5.2 (3.9–7.8)	98.2 (81.3–109.2)	39.5 (33.5–45.6)	0.18 (0.15–0.21)	0.46 (0.42–0.48)
No (*n* = 16)	32.3 (20.0–51.4)	7.2 (5.1–9.4)	101.4 (86.6–117.9)	41.4 (35.4–47.2)	0.22 (0.16–0.29)	0.48 (0.45–0.51)
Visual impairment at diagnosis						
Yes (*n* = 13)	32.3 (20.0–55.3)	8.1 (5.5–9.6)	108.8 (93.3–116.7)	44.7 (37.9–46.9)	0.21 (0.16–0.27)	0.47 (0.43–0.51)
No (*n* = 11)	31.0 (21.7–36.7)	5.4 (3.8–8.9)	97.9 (75.4–107.3)	38.5 (31.3–43.1)	0.18 (0.12–0.29)	0.47 (0.45–0.49)
Hypopituitarism at diagnosis						
Yes (*n* = 11)	36.7 (21.0–55.0)	8.6 (5.9–9.4)	99.5 (89.1–119.2)	40.6 (36.4–47.5)	0.22 (0.16–0.29)	0.47 (0.44–0.49)
No (*n* = 13)	30.0 (20.0–35.8)	4.9 (3.9–7.9)	98.5 (74.5–110.4)	39.6 (30.3–46.4)	0.18 (0.13–0.26)	0.47 (0.43–0.53)
Cavernous sinus invasion						
Yes (*n* = 10)	28.3 (16.8–41.4)	6.1 (5.1–9.6)	100.9 (84.1–140.6)	40.6 (34.5–56.1)	0.24 (0.15–0.42)	0.47 (0.42–0.49)
No (*n* = 14)	32.3 (27.5–44.3)	5.8 (4.2–9.0)	98.7 (83.7–110.1)	40.4 (34.6–45.7)	0.19 (0.15–0.22)	0.48 (0.44–0.53)
Ki-67						
<3% (*n* = 19)	32.3 (21.7–40.7)	5.9 (4.3–9.4)	97.9 (79.3–110.9)	39.5 (32.6–46.4)	0.17 (0.15–0.25)	0.48 (0.44–0.51)
≥ 3% (*n* = 5)	20.3 (17.2–63.8)	6.3 (4.7–14.4)	109.9 (100.7–138.8)	44.7 (41.4–57.3)	0.23 (0.21–0.35)	0.47 (0.37–0.48)
Trouillas classification grade						
1a (*n* = 11)	32.3 (29.7–40.7)	5.7 (4.3–8.6)	97.9 (73.5–108.8)	39.6 (29.3–45.5)	0.17 (0.15–0.22)	0.48 (0.44–0.53)
1b (*n* = 3)	40.6 (20.3–.)	8.9 (3.9–.)	109.9 (97.9–.)	44.7 (40.2–.)	0.22 (0.19–.)	0.47 (0.41–.)
2a (*n* = 8)	35.8 (18.4–50.9)	7.4 (4.5–9.8)	93.8 (80.9–132.7)	37.4 (33.2–51.8)	0.17 (0.14–0.40)	0.48 (0.44–0.49)
2b (*n* = 2)	17.2 (15.3–.)	5.9 (5.6–.)	133.4 (103.3–.)	55.4 (42.7–.)	0.35 (0.29–.)	0.40 (0.33–.)
Re-operation						
Yes (*n* = 5)	40.7 (35.3–76.0)^a^	8.8 (5.9–11.6)	89.1 (72.2–97.9)	36.3 (28.2–40.4)	0.16 (0.13–0.21)	0.49 (0.48–0.53)^a^
No (*n* = 19)	31.0 (19.7–36.7)	5.7 (4.3–9.4)	107.3 (87.1–119.2)	43.1 (36.4–47.5)	0.19 (0.15–0.29)	0.46 (0.43–0.49)

Microvessel density, total microvessel area and vascular architecture parameters among the cohort of 24 patients with pituitary neuroendocrine tumours (PitNETs) according to different clinico-pathological features. Data are shown as median (interquartile range). *P* values were non-significant for all comparisons

*HPF* high-power field, *MVD* microvessel density, *TMVA* total microvessel area

^a^*p* value comprised between 0.05 and 0.1 (Mann–Whitney *U* tests were used for all comparisons, except for the Trouillas classification grades where Kruskal–Wallis test was applied)

**Fig. 2:**
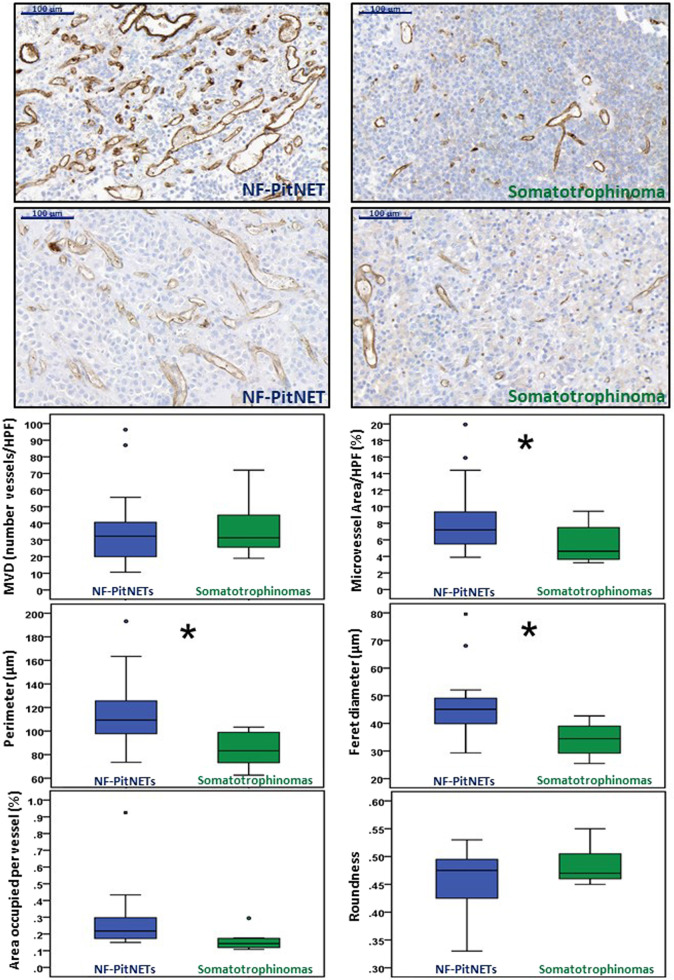
Angiogenesis in NF-PitNETs and somatotrophinomas. Microvessel density (MVD) and vasculature architecture parameters differences between human non-functioning pituitary neuroendocrine tumours (NF-PitNETs) and somatotrophinomas are shown. NF-PitNET (*n* = 16) and somatotrophinoma (*n* = 8) tissue sections were stained for CD31. CD31 positive vessels were counted in three different high-power fields (HPF) to obtain MVD (number of vessels/HPF). CD31-stained ×20 magnification fields were analysed with ImageJ and vessel contour was manually traced in order to obtain the vasculature architecture parameters: total microvessel area, area occupied per vessel, vessel perimeter, vessel Feret’s diameter and roundness (vessel roundness correspond to a value comprised between 0 and 1, with 1 = perfect circle). Representative images of vessels from two NF-PitNETs and two somatotrophinomas are shown (×20). Scale bar 100 µm. *p < 0.05, **p < 0.01, ***p < 0.001 (Mann–Whitney *U* test)

Microvessel density did not differ among NF-PitNETs and somatotrophinomas, but the total microvessel area, vessel perimeter and Feret’s diameter were significantly higher in NF-PitNETs. The area occupied per vessel tended (*p* = 0.097) to also be higher in NF-PitNETs than in somatotrophinomas (Fig. 2). There were no differences between untreated somatotrophinomas (*n* = 2) and those treated with somatostatin analogues before operation (*n* = 6) regarding microvessel density, total microvessel area, vessel perimeter, Feret’s diameter, area occupied per vessel or microvessel roundness (data not shown). There were also no differences concerning the vasculature density and morphology between sparsely granulated (*n* = 5) and densely granulated (*n* = 3) somatotrophinomas (data not shown).

**Fig. 3:**
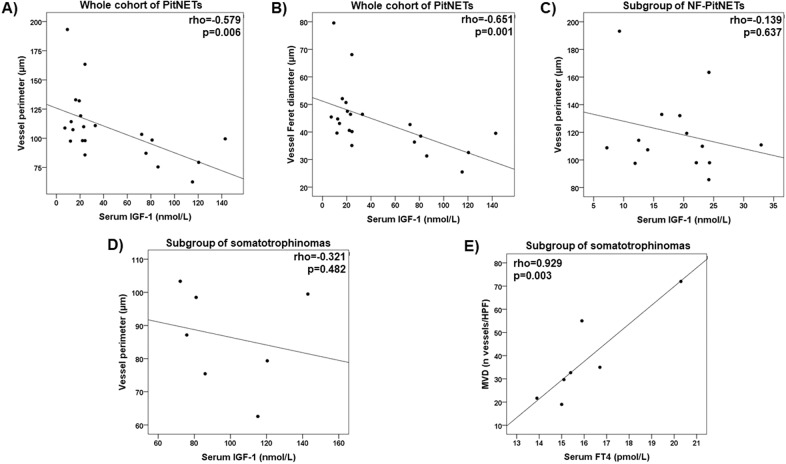
Serum pituitary hormone levels and vessel parameters in PitNETs. **a** Correlation between serum IGF-1 levels and vessel perimeter within the whole cohort of PitNETs. **b** Correlation between serum IGF-1 levels and Feret’s diameter within the whole cohort of PitNETs. **c** Correlation between serum IGF-1 levels and vessel perimeter within NF-PitNETs. **d** Correlation between serum IGF-1 and vessel perimeter within somatotrophinomas. **e** Correlation between serum-free FT4 levels and MVD within somatotrophinomas. *P* values were determined by the Spearman’s correlation coefficient rho. FT4 free thyroxine, HPF high-power field, IGF-1 insulin-like growth factor 1, MVD microvessel density, NF-PitNET non-functioning pituitary neuroendocrine tumour, PitNET pituitary neuroendocrine tumour

Data regarding the serum pituitary hormone levels in our cohort of PitNETs are shown in the Supplementary Table 1. Overall, there were no correlations between microvessel density, microvessel area or vascular architecture parameters, and pituitary hormone levels in our cohort of PitNETs (data not shown), except for the significant negative correlation between serum insulin-like growth factor 1 (IGF-1) levels and both perimeter and Feret’s diameter in the whole PitNET cohort (Fig. 3a, b). This correlation was not observed within the subgroups of NF-PitNETs (Fig. 3c) or somatotrophinomas (Fig. 3d). Within the subgroup of somatotrophinomas, free thyroxine levels correlated with microvessel density (rho = 0.929; *p* = 0.003) (Fig. 3e).

### PitNET-derived cytokine secretome and angiogenesis in PitNET patients

**Fig. 4:**
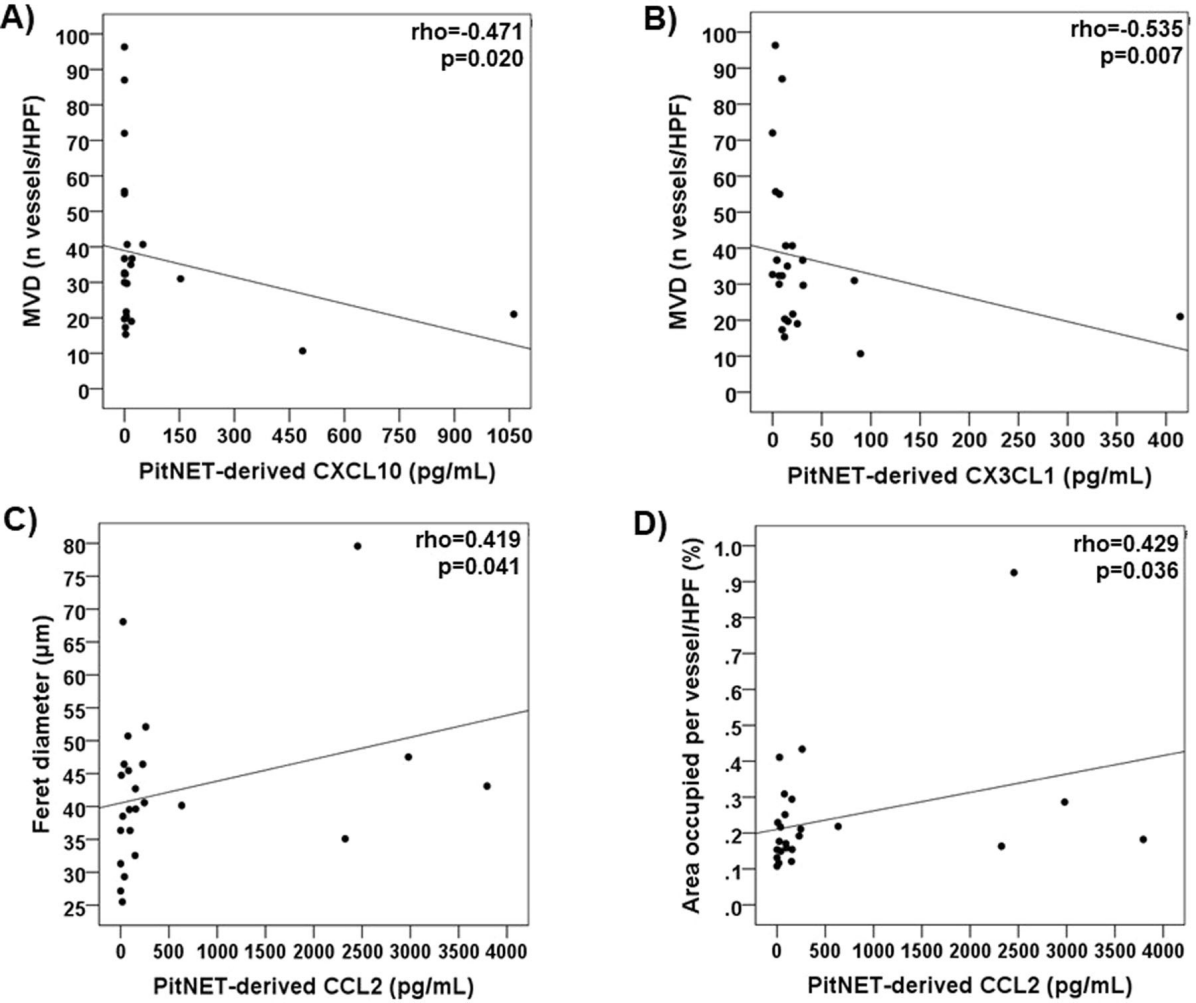
PitNET-derived cytokines and vessel perimeters in the whole cohort of 24 PitNETs. **a** Correlation between PitNET-derived CXCL10 and microvessel density. **b** Correlation between PitNET-derived CX3CL1 and microvessel density. **c** Correlation between PitNET-derived CCL2 and Feret’s diameter. **d** Correlation between PitNET-derived CCL2 and area occupied per vessel. *P* values were determined by the Spearman’s correlation coefficient rho. HPF high-power field, MVD microvessel density, PitNET pituitary neuroendocrine tumour

PitNET-derived cytokine secretome data from our PitNET cohort has been previously reported [9]. We observed a negative correlation between microvessel density and the levels of PitNET-derived CXCL10 (rho = −0.471; *p* = 0.020) and CX3CL1 (rho = −0.535; *p* = 0.007) (Fig. 4a, b). There were also significant correlations between CCL2 levels and the Feret’s diameter (rho = 0.419; *p* = 0.041) and area occupied per vessel (rho = 0.429; *p* = 0.036) (Fig. 4c, d). There were no other significant correlations between PitNET-derived cytokines and microvessel density, area or other vessel morphology parameters (Supplementary Table 2), even for proteins with recognised angiogenic properties such as VEGF-A, IL-8 and FGF-2 [12, 25].

### PitNET-infiltrating immune cells and angiogenesis in PitNET patients

**Fig. 5:**
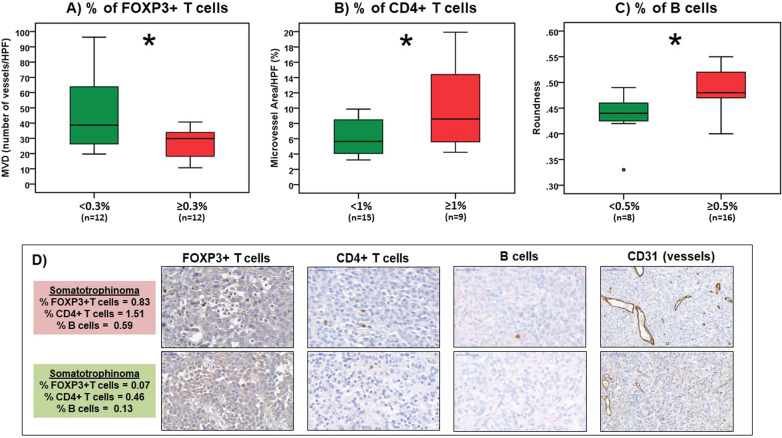
Tumour-infiltrating immune cells and angiogenesis in PitNETs. Microvessel density (MVD) and vascular architecture parameters in pituitary neuroendocrine tumours (PitNETs) with lower versus higher amounts of **a** FOXP3+ T cells, **b** CD4+ T cells and **c** B cells. The PitNET-infiltrating immune cell thresholds considered here were the same as those previously published in [9]. **d** Representative images of a tumour with lower versus a sample with higher amounts of FOXP3+ T, CD4+ T and B cells are shown (×20); scale bar: 100 µm. *p < 0.05, **p < 0.01, ***p < 0.001 (Mann–Whitney *U* test)

PitNET-infiltrating immune cells data from our PitNET cohort have been described in detail elsewhere [9]. Microvessel density was higher in PitNETs with fewer FOXP3+ T cells (Fig. 5a and Supplementary Table 3). PitNETs with more CD4+ T cells were significantly associated with increased microvessel area (Fig. 5b). The M2:M1 macrophage ratio correlated positively with microvessel area (rho = 0.461; *p* = 0.023) (Supplementary Table 3). There were no correlations between any of the immune cells studied and vessel architecture parameters, except for B cells which were associated with increased vessel roundness (Fig. 5c).

NF-PitNETs with a higher content of infiltrating CD4+ T lymphocytes (≥1%) had increased microvessel density (40.7 (32.3–87.0) vs 20.3 (16.3–34.5) vessels/HPF; *p* = 0.044), increased total microvessel area (8.9 (6.0–15.9) vs 5.9 (4.8–8.5) % of microvessel area/HPF; *p* = 0.032) and were more round (0.49 (0.48–0.53) vs 0.43 (0.41–0.47); *p* = 0.004) than NF-PitNETs with CD4+ T cell counts <1%. The vessels were also more round in NF-PitNETs with higher contents of infiltrating B cells (0.48 (0.44–0.51) vs 0.43 (0.38–0.46); *p* = 0.048). Vessels from NF-PitNETs with more infiltrating FOXP3+ T cells (≥0.3%) had increased perimeter (132.1 (97.9–163.4) vs 107.3 (87.4–112.5) µm; *p* = 0.029) and Feret’s diameter (50.7 (40.2–68.1) vs 43.1 (35.7–45.9) µm; *p* = 0.033) than those NF-PitNETs with fewer FOXP3+ T cells (Supplementary Table 4). The somatotrophinomas with an increased amount of macrophages (≥6%) had larger microvessels than those with fewer infiltrating macrophages, namely higher perimeter, higher Feret’s diameter and also each vessel occupied an increased area (Supplementary Table 5).

## Discussion

**Fig. 6:**
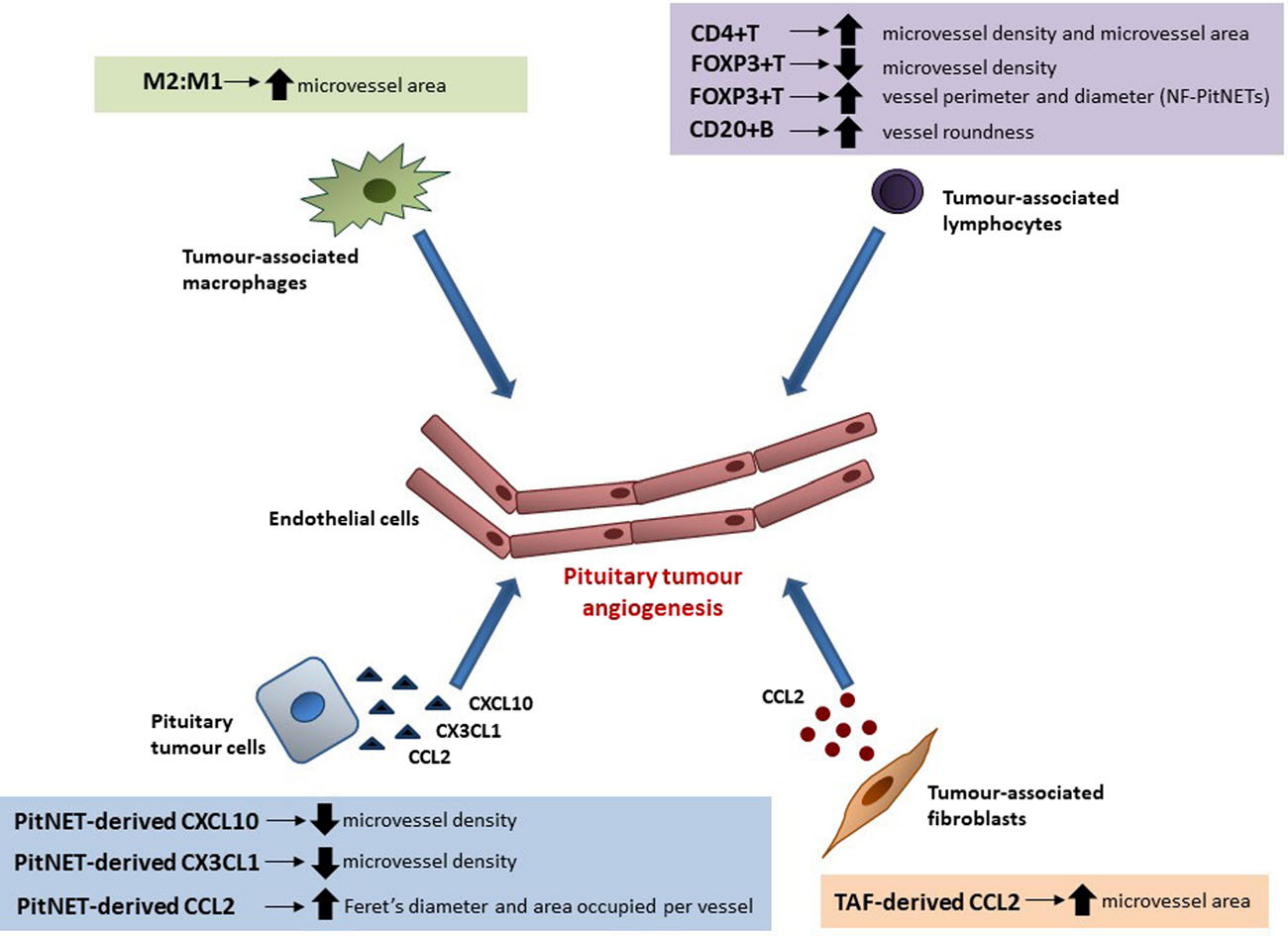
Modulation of the PitNET angiogenesis by different TME elements. Different proteins are secreted by the pituitary tumour cells into the tumour microenvironment (TME) of PitNETs, and some of these may be able to influence and modulate the pituitary tumour angiogenesis, such as CCL2, CXCL10 and CX3CL1. Proteins released by non-neoplastic infiltrating immune cells present within the TME of PitNETs, such as macrophages, CD4+ T cells, FOXP3+ T cells, B cells, may further influence the pituitary tumour neovascularisation. In addition, PitNET-associated tumour-associated fibroblasts (TAFs) are also a source of cytokines and chemokines, including CCL2, which may further modulate the angiogenesis of PitNETs [10]. CD4+ T, CD4+ T cells; CD20+ B, CD20+ B lymphocytes; FOXP3+ T, FOXP3+ T regulatory cells; M2:M1 M2 and M1 macrophage ratio, NF-PitNETs non-functioning pituitary neuroendocrine tumours, PitNET pituitary neuroendocrine tumour, TAF pituitary tumour-associated fibroblast

In this study, using primary culture cytokine data and tumour-infiltrating immune cell immunohistochemical data from a cohort of 24 human PitNETs, we found that certain TME elements may be associated with angiogenesis in PitNETs. Significant associations reported in this study, as well as the angiogenesis-related findings from our previously published data on PitNET-associated fibroblasts [10], are summarised in Fig. 6. Specifically, while these are simple associations at present, we noted that M2-like macrophages emerge as potentially playing a relevant role in PitNET angiogenesis; B, CD4+ T and FOXP3+ T lymphocytes, together with PitNET-derived CCL2, CXCL10 and CX3CL1, may also play a modulatory role in PitNET neovascularisation.

Angiogenesis provides tumour cells with energy supply and oxygen necessary for tumour growth, and increased requirements are needed for aggressive tumour growth [16, 25]. Angiogenesis has been previously studied in the neoplastic and NP [12, 16], but little is known regarding the possible role of different cellular and non-cellular TME elements in PitNET angiogenesis [24]. We previously reported that PitNET-infiltrating M2-like macrophages [9] and CCL2 derived from PitNET-associated fibroblasts may play a modulatory role in the angiogenesis of PitNETs [10]. In this study, we expand on these and other TME elements potentially contributing to the modulation of PitNET neovascularisation.

PitNETs are less vascularised than NP [13, 19, 36], and many studies excluded an association between vessel density and PitNET proliferation or invasiveness, indicating that other factors underlie the invasive potential of PitNETs [12, 16, 18, 19, 36, 37], consistent with our own observations. Turner et al. did not find differences in microvessel density between invasive and non-invasive somatotrophinomas and corticotrophinomas, although invasive prolactinomas were significantly more vascular than non-invasive ones [17]. Overall, these observations raise uncertainty regarding the role of angiogenesis in PitNETs, as opposed to other malignancies in which neovascularisation correlates with tumour growth, invasion and metastasis [12, 25, 38], including in meningiomas where an increased microvessel density was observed for higher grade [39] and recurring meningiomas [40]. However, the lack of increased angiogenesis in PitNETs in comparison to NP, and the lack of association between vascularisation and PitNET aggressiveness, may underlie the low growth rate and benign nature of PitNETs which uncommonly metastasise [12, 16, 18]. This apparent paradox can be partially explained by the lower oxygen consumption rate of PitNET cells. Tumour vessels can themselves be hypoxic and carry little oxygen, or can have oscillating rather than directed blood flow and thus be ineffective in transporting oxygen. Moreover, tumour cells are known to tolerate oxygen deprivation and be resistant to apoptosis under hypoxia, which allows for increased intercapillary distance [41]. In contrast, increased microvessel density was described for pituitary carcinomas as compared to PitNETs and NP [13, 17–22], consistent with the fact that distant metastasis depends on angiogenesis [18]. These data suggest that angiogenesis may be a relevant mechanism at least for highly aggressive metastatic pituitary tumours.

We observed that somatotrophinomas were less vascularised and have smaller vessels than NF-PitNETs, in line with previous observations [18, 42], although other studies reported no angiogenesis differences between PitNET subtypes [11, 13, 17]. The differences we observed are unlikely to be due to pre-operative somatostatin analogue treatment, as there were no angiogenic differences between untreated and pre-treated somatotrophinomas, in line with previous reports showing that neither somatostatin analogues nor dopamine agonists affect PitNET microvessel density [17, 18, 23]. We found a negative correlation between serum IGF-1 and perimeter and Feret’s diameter within the whole cohort of PitNETs. This could be overlooked as IGF-1 hypersecretion resulting in smaller vessel as an explanation for the vasculature differences between NF-PitNETs and somatotrophinomas, a somewhat paradoxical speculation with the recognised angiogenic properties of IGF-1 [43, 44]. Considering that this correlation was lost when NF-PitNETs and somatotrophinomas were analysed separately, it is unlike that IGF-1 on its own explains the vascular differences within, and possibly between, somatotrophinomas and NF-PitNETs, and this observed correlation for the whole PitNET cohort occurred only because somatotrophinomas, which have by definition elevated IGF-1 levels, had smaller vessels than NF-PitNETs. In our cohort of PitNETs, as well as within NF-PitNET or somatotrophinoma subgroups, we did not find significant correlations between serum prolactin levels and microvessel density or vessel morphology parameters, although prolactin has recognised angiogenic properties [45, 46], and previous studies showed that prolactinomas may have higher microvessel density compared to other PitNET types [11, 13, 17] which could be, at least in part, due to prolactin hypersecretion. Within somatotrophinomas, free thyroxine levels correlated with microvessel density, consistent with the angiogenic properties of thyroid hormones [47, 48].

In our study, PitNET microvessel area correlated positively with the M2:M1 macrophage ratio, which together with data from a recent study showing more M2-like macrophages in rat prolactinomas than in NP, and that tumour M2-like macrophage content increase as capillaries became more tortuous and of increased calibre [49], support an angiogenic role for M2-like macrophages in PitNETs, as shown for other cancers [12, 14, 25, 26, 33, 38, 50, 51]. Increased microvessel density and area were also seen in PitNETs with more CD4+ T cells, whereas PitNETs with more FOXP3+ T cells had decreased microvessel density. Within NF-PitNETs, increased amounts of infiltrating CD4+ and FOXP3+ T cells were associated with bigger vessels, while in somatotrophinomas larger vessels correlated with more macrophages. The crosstalk between tumour and immune cells in hypoxic and cytokine-rich TME result in the induction of pro-angiogenic behaviour in both cell types and thus may promote tumour neovascularisation [38, 52]. Macrophages, CD4+ T and FOXP3+ T cells are strong promoters of angiogenesis in tumours [38, 52, 53], explaining some of our findings. However, the immunosuppressive activity of FOXP3+ T cells on macrophages and CD4+ T cells may impair their angiogenic functions, resulting in angiogenesis suppression [54], possibly explaining why PitNETs with more FOXP3+ cells associated with a lower vessel density. Overall, these data suggest that immune cells may influence PitNET angiogenesis, particularly macrophages and T lymphocytes, both active sources of angiogenic cytokines and growth factors in the TME [38, 53, 55]. The relatively modest infiltration of immune cell seen in PitNETs [9, 56], and thus the lower degree of tumour inflammation resulting in lower pro-inflammatory and pro-angiogenic factors, may explain the reduced angiogenesis in PitNETs when compared to NP [13, 19, 36], as well as to pituitary carcinomas [13, 17–22] or other malignant tumours [25, 38].

Tumour cell-derived cytokines and growth factors are able to directly modulate angiogenesis and affect tumour vessel morphology [25]. PitNET-derived CCL2 levels were associated with bigger vessels (higher Feret’s diameter and higher area occupied per vessel), suggesting a possible role for CCL2 in the modulation of PitNET angiogenesis. These findings are in line with the recognised pro-angiogenic properties of CCL2 [57, 58], and also with data from another study showing that CCL2 released by pituitary tumour-associated fibroblasts correlated with the PitNET microvessel area [10]. We also observed a negative correlation between microvessel density and the levels of CXCL10 and CX3CL1 released by pituitary tumour cells. These two chemokines have been involved in the regulation of tumoural neovascularisation, CXCL10 being usually described as an inhibitor of angiogenesis [59], whereas CX3CL1 has pro-angiogenic effects [60, 61]. Nevertheless, most of our data correlating PitNET-derived cytokines and angiogenic parameters were non-significant, including those for recognised angiogenic factors such as VEGF-A [11, 62], FGF-2 [12, 25] and IL-8 [63], possibly implying that cytokines released by pituitary tumour cells per se may play a limited direct effect in the tumour angiogenesis. Alternatively, other factors, such as the hormonal milieu within PitNETs, or angiogenic cytokines derived from non-neoplastic cells within the TME, may be more relevant for PitNET angiogenesis regulation than factors secreted by PitNET cells, as described for other cancers [38, 53, 55]. Nevertheless, the PitNET-derived chemokines may have an indirect angiogenic role by promoting the chemoattraction of immune cells into the TME of PitNETs [9], which in turn may exert a direct and perhaps more prominent pro-angiogenic effect within the tumour tissue. In addition to the pituitary tumour cell and immune cell-derived angiogenic compounds, secreted proteins from pituitary tumour-associated fibroblasts, namely CCL2 as previously shown [10], may further contribute for PitNET angiogenesis, as described in other cancers [15, 64, 65].

Angiogenesis is commonly evaluated by immunohistochemistry assessing microvessel density and vasculature morphology by examining CD31- or CD34-stained tissue sections in image analyser systems [11, 12, 41], a method we used in our study. However, the immunohistochemical assessment of angiogenesis has a number of shortcomings that can explain some inconsistencies reported in previous studies and represent limitations to our own study. Firstly, as in any tumour, PitNETs have a complex biology and irregular geometry of the vascular system, which vary from case to case and within the different pituitary tumour types [11, 16]. Secondly, some tumours (including PitNETs) have lower microvessel density than the corresponding normal tissues, hence the assessment of microvessel density may not be sufficient to reveal the functional or angiogenic status of a tumour [41]. Thirdly, it is important to take into account vessel topography in the selection of the fields to assess, differentiating vessels into those supplying invading tumour edges, those serving the inner tumour area and those in the peripheral tumour areas usually composed of capillaries with endothelial cells derived from pre-existing vessels [41]. Fourthly, attention should be paid to vessel diameter, where tumours with high metabolic rate usually have small vessel diameter and high vascular density; in contrast, tumours with low metabolic rate have larger vessels and a relatively low vascular density. Fifthly, variability in the results can be also due to the lack of standardised protocols in manual or automated vessel counting or due to technical aspects such as observer subjectivity, choice to count vessels in randomly chosen fields or hot spot areas (the method we followed in this study), field magnification, and the selection of the endothelial marker to use [41]. We included a well-characterised but small cohort of cases in our study, which adds further limitations to our study, as it may provide insufficient statistical power to detect significant differences, particularly if we take in consideration that cytokine concentrations and infiltrating immune cells had substantial inter-individual variability. Therefore, some of the negative findings we observed may not reflect the lack of an association, but instead an insufficient sample size for some of the comparative analyses we conducted here. Lastly, taking into account the exploratory nature of this study, the first of its kind exploring the role of different TME elements in pituitary tumour angiogenesis, we analysed various immune cells and cytokines. This resulted in multiple comparisons, which may constitute another statistical limitation to our study. Hence, further studies using more samples, as well as including functional experiments with cell lines or animal models, are needed to validate some of the significant findings (and also the negative data) from our exploratory study.

## Conclusions

Our data suggest that cellular and non-cellular TME elements may play a modulatory role in tumoural angiogenesis in PitNETs. While tumour-derived cytokines, such as CCL2, CXCL10 and CX3CL1, or TAF-derived cytokines such as CCL2, may have some direct influence on angiogenesis, our data suggest that tumour-infiltrating immune cells, particularly M2 macrophages, CD4+ T and FOXP3+ T and B cells, may also have an angiogenic impact.

## Supplementary information

Supplemental Table 1

Supplemental Table 2

Supplemental Table 3

Supplemental Table 4

Supplemental Table 5
