# Supplementary material for: The role of the tumour microenvironment in the angiogenesis of pituitary tumours
Source: Endocrine. 2020 Sep 18;70(3):593–606. doi: 10.1007/s12020-020-02478-z (PMC7674353; doi:10.1007/s12020-020-02478-z)
Supplement: Supplementary file 1 — Supplemental Table 1 [file 12020_2020_2478_MOESM1_ESM.docx]

| **Pituitary hormone** | **Overall cohort of PitNETs** | **NF-PitNETs** | **Somatotrophinomas** |
| --- | --- | --- | --- |
| **Serum IGF-1 (nmol/L)** | 24.2 (17.4-74.9) | 22.1 (12.5-24.2) | 81.0 (74.0-131.7) |
| **Random serum GH (µg/L)** | 0.5 (0.1-5.9) | 0.1 (0.1-0.7) | 15.2 (4.7-62.7) |
| **Serum prolactin (mU/L)** | 414.5 (261.0-810.5) | 349.0 (244.0-625.0) | 761.0 (234.0-3058.0) |
| **Serum TSH (µU/mL)** | 1.5 (0.7-2.1) | 1.9 (1.3-2.4) | 0.6 (0.1-1.0) |
| **Serum FT4 (pmol/L)** | 13.3 (10.4-15.8) | 12.7 (9.5-13.5) | 15.4 (14.5-16.3) |
| **Serum LH (U/L)** | 3.9 (1.2-7.0) | 3.3 (1.1-10.2) | 5.3 (1.9-6.5) |
| **Serum FSH (U/L)** | 8.4 (4.3-16.8) | 10.5 (6.4-32.7) | 5.0 (3.9-6.6) |
| **Basal plasma cortisol (nmol/l)** | 304.5 (174.3-469.3) | 279.0 (119.0-481.0) | 330.0 (207.0-552.0) |

**Supplemental Table 1:** **Serum pituitary hormones in the whole cohort of 24 PitNETs**

Blood samples for the measurement of serum pituitary hormones were routinely taken at diagnosis and before the pituitary surgery, and assayed in a certified National Health Service laboratory. Data are shown as median (interquartile range) per pituitary hormone for the whole cohort of 24 PitNETs, and for the subgroups of NF-PitNETs (n=16) and somatotrophinomas (n=8). ACTH, adrenocorticotropic hormone; FSH, follicle-stimulating hormone; FT4, free thyroxine; GH, growth hormone; IGF-1, insulin-like growth factor-1; LH, luteinising hormone; NF-PitNETs; non-functioning pituitary neuroendocrine tumours; PitNETs, pituitary neuroendocrine tumours; TSH, thyroid-stimulating hormone.
