# Supplementary material for: The role of the tumour microenvironment in the angiogenesis of pituitary tumours
Source: Endocrine. 2020 Sep 18;70(3):593–606. doi: 10.1007/s12020-020-02478-z (PMC7674353; doi:10.1007/s12020-020-02478-z)
Supplement: Supplementary file 2 — Supplemental Table 2 [file 12020_2020_2478_MOESM2_ESM.docx]

|  | **MVD** | **TMVA** | **Perimeter** | **Feret’s diameter** | **Area per vessel** | **Roundness** |
| --- | --- | --- | --- | --- | --- | --- |
| **PitNET-derived cytokine data** *[Spearman’s correlation rho (p)]*  IL-8  CCL2  CCL3  CCL4  CXCL10  CCL22  CXCL1  CX3CL1  FGF-2  IL-6  PDGF-AA  VEGF-A | -0.126 (*p*=0.557)  -0.122 (*p*=0.569)  -0.132 (*p*=0.538)  -0.182 (*p*=0.394)  -0.471 (***p*=0.020**)  -0.266 (*p*=0.208)  -0.166 (*p*=0.438)  -0.535 (***p*=0.007**)  0.015 (*p*=0.943)  -0.338 (*p*=0.106)  -0.271 (*p*=0.201)  -0.132 (*p*=0.538) | 0.203 (*p*=0.340)  0.211 (*p*=0.323)  0.154 (*p*=0.473)  0.102 (*p*=0.636)  -0.212 (*p*=0.321)  -0.178 (*p*=0.406)  -0.046 (*p*=0.829)  -0.283 (*p*=0.180)  -0.300 (*p*=0.155)  0.036 (*p*=0.866)  0.088 (*p*=0.683)  -0.047 (*p*=0.827) | 0.349 (*p*=0.095)  0.358 (*p*=0.086)  0.285 (*p*=0.177)  0.220 (*p*=0.302)  0.202 (*p*=0.344)  0.115 (*p*=0.593)  0.086 (*p*=0.689)  0.260 (*p*=0.219)  -0.323 (*p*=0.124)  0.347 (*p*=0.097)  0.359 (*p*=0.085)  0.102 (*p*=0.634) | 0.388 (*p*=0.061)  0.419 (***p*=0.041**)  0.353 (*p*=0.091)  0.275 (p=0.194)  0.252 (*p*=0.234)  0.098 (*p*=0.648)  0.108 (*p*=0.617)  0.266 (*p*=0.208)  -0.305 (*p*=0.147)  0.323 (*p*=0.139)  0.389 (*p*=0.060)  0.111 (*p*=0.604) | 0.372 (*p*=0.072)  0.429 (***p*=0.036**)  0.303 (*p*=0.150)  0.302 (*p*=0.152)  0.282 (*p*=0.181)  0.156 (*p*=0.468)  0.171 (*p*=0.425)  0.290 (*p*=0.170)  -0.225 (*p*=0.291)  0.346 (*p*=0.129)  0.305 (*p*=0.147)  0.066 (*p*=0.759) | -0.037 (*p*=0.863)  0.000 (*p*=1.000)  -0.012 (*p*=0.954)  0.057 (*p*=0.792)  -0.097 (*p*=0.651)  -0.152 (*p*=0.478)  0.064 (*p*=0.766)  -0.106 (*p*=0.621)  0.223 (*p*=0.295)  -0.149 (*p*=0.488)  -0.025 (*p*=0.908)  -0.024 (*p*=0.912) |

**Supplemental Table 2: Correlation between pituitary tumour-derived cytokines and angiogenesis in PitNETs**

PitNET-derived cytokines and angiogenesis data are shown for the whole cohort of 24 PitNETs. Microvessel density (MVD) is expressed in vessels/HPF; total microvessel area (TMVA) is expressed in % of the HPF; perimeter and Feret’s diameter are expressed in µm; area per vessel is expressed in % of the HPF; vessel roundness correspond to a value comprised between 0 and 1 (1=perfect circle). The correlations between the continuous variables were determined by the Spearman’s correlation coefficient rho. FGF-2, fibroblast growth factor-2; HPF, high power field; IL, interleukin; MVD, microvessel density; PDGF-AA, platelet-derived growth factor-AA; PitNET, pituitary neuroendocrine tumour; TMVA, total microvessel area; VEGF-A, vascular endothelial growth factor-A.
