# Supplementary material for: The role of the tumour microenvironment in the angiogenesis of pituitary tumours
Source: Endocrine. 2020 Sep 18;70(3):593–606. doi: 10.1007/s12020-020-02478-z (PMC7674353; doi:10.1007/s12020-020-02478-z)
Supplement: Supplementary file 3 — Supplemental Table 3 [file 12020_2020_2478_MOESM3_ESM.docx]

|  | **MVD** | **TMVA** | **Perimeter** | **Feret’s diameter** | **Area per vessel** | **Roundness** |
| --- | --- | --- | --- | --- | --- | --- |
| **PitNET-infiltrating macrophages** *[Median (IQR)]*  < 6% (n=17)  ≥ 6% (n=7) | 30.0 (20.0-35.8)  40.7 (31.0-55.7)  *p*=0.184 | 5.4 (4.1-9.0)  8.8 (5.7-9.4)  *p*=0.228 | 98.5 (77.4-125.7)  99.5 (89.1-107.3)  *p*=0.448 | 40.6 (31.9-49.1)  40.2 (36.3-43.1)  *p*=0.408 | 0.19 (0.14-0.29)  0.18 (0.16-0.23)  *p*=0.495 | 0.47 (0.43-0.52)  0.47 (0.46-0.49)  *p*=0.711 |
| **PitNET-infiltrating CD8+ T cells** *[Median (IQR)]*  < 1% (n=6)  ≥ 1% (n=18) | 27.0 (18.6-44.4)  32.5 (20.8-44.3)  *p*=0.441 | 7.2 (4.2-8.6)  5.8 (4.8-9.6)  *p*=0.557 | 103.6 (95.7-123.9)  98.7 (78.4-115.5)  *p*=0.522 | 43.0 (37.9-51.8)  39.9 (32.2-46.7)  *p*=0.432 | 0.21 (0.17-0.29)  0.18 (0.14-0.29)  *p*=0.967 | 0.45 (0.39-0.48)  0.48 (0.45-0.52)  *p*=0.064 |
| **PitNET-infiltrating CD4+ T cells** *[Median (IQR)]*  < 1% (n=15)  ≥ 1% (n=9) | 30.0 (19.7-36.7)  36.7 (26.7-63.8)  *p*=0.152 | 5.7 (3.9-8.8)  8.6 (5.3-15.2)  ***p*=0.035** | 99.5 (85.7-110.9)  97.9 (88.4-116.7)  *p*=0.724 | 39.5 (35.1-46.4)  40.6 (36.1-46.1)  *p*=0.665 | 0.18 (0.15-0.25)  0.22 (0.15-0.29)  *p*=0.878 | 0.45 (0.42-0.49)  0.48 (0.47-0.52)  *p*=0.051 |
| **PitNET-infiltrating B cells** *[Median (IQR)]*  < 0.5% (n=8)  ≥ 0.5% (n=16) | 26.3 (16.4-49.4)  33.7 (23.2-40.7)  *p*=0.292 | 5.9 (4.5-9.3)  6.0 (4.4-9.3)  *p*=0.467 | 103.4 (91.4-150.3)  97.9 (76.4-113.1)  *p*=0.098 | 41.3 (36.9-62.7)  40.4 (31.6-46.2)  *p*=0.174 | 0.18 (0.16-0.36)  0.20 (0.14-0.28)  *p*=0.386 | 0.44 (0.42-0.46)  0.48 (0.47-0.53)  ***p*=0.015** |
| **PitNET-infiltrating neutrophils** *[Median (IQR)]*  < 0.5% (n=13)  ≥ 0.5% (n=11) | 32.7 (21.0-55.3)  31.0 (17.3-40.7)  *p*=0.522 | 5.6 (4.1-9.4)  6.0 (5.4-8.9)  *p*=0.720 | 99.5 (83.2-110.4)  97.9 (85.7-132.1)  *p*=0.225 | 39.5 (34.4-45.9)  40.6 (35.1-50.7)  *p*=0.259 | 0.18 (0.14-0.24)  0.21 (0.15-0.31)  *p*=0.257 | 0.47 (0.45-0.49)  0.48 (0.43-0.53)  *p*=0.927 |
| **PitNET-infiltrating FOXP3+ T cells** *[Median (IQR)]*  < 0.3% (n=12)  ≥ 0.3% (n=12) | 38.7 (24.0-67.9)  29.8 (17.7-34.4)  ***p*=0.021** | 8.4 (5.7-9.4)  5.2 (3.9-8.2)  *p*=0.182 | 98.9 (86.6-110.3)  110.7 (81.3-132.7)  *p*=0.312 | 40.1 (35.4-45.3)  41.4 (33.5-51.8)  *p*=0.304 | 0.18 (0.16-0.23)  0.21 (0.13-0.39)  *p*=0.198 | 0.48 (0.44-0.49)  0.47 (0.42-0.52)  *p*=0.659 |
| **Immune cell ratios** *[Spearman’s correlation rho (p value)]*  M2:M1  CD8:CD4  CD8:FOXP3  CD68:FOXP3 | 0.230 (*p*=0.280)  -0.013 (*p*=0.950)  0.353 (*p*=0.091)  0.323 (*p*=0.124) | 0.461 (***p*=0.023**)  -0.027 (*p*=0.902)  0.130 (*p*=0.546)  0.194 (*p*=0.364) | 0.139 (*p*=0.517)  -0.096 (*p*=0.655)  -0.254 (*p*=0.231)  -0.063 (*p*=0.770) | 0.140 (*p*=0.515)  -0.098 (*p*=0.650)  -0.262 (*p*=0.216)  -0.096 (*p*=0.655) | 0.083 (*p*=0.701)  -0.142 (*p*=0.509)  -0.261 (*p*=0.218)  -0.048 (*p*=0.824) | 0.141 (*p*=0.511)  -0.104 (*p*=0.629)  0.323 (*p*=0.124)  0.159 (*p*=0.459) |

**Supplemental Table 3: Correlation between infiltrating immune cells and angiogenesis in PitNETs**

PitNET-infiltrating immune cell and angiogenesis data are shown for the whole cohort of 24 PitNETs. Microvessel density (MVD) is expressed in vessels/HPF; total microvessel area (TMVA) is expressed in % of the HPF; perimeter and Feret’s diameter are expressed in µm; area per vessel is expressed in % of the HPF; vessel roundness correspond to a value comprised between 0 and 1 (1=perfect circle). The PitNET-infiltrating immune cell thresholds considered here were the same as those previously published in [9]. The correlations between immune cell ratios and vessel parameters were determined by the Spearman’s correlation coefficient rho. Mann Whitney U tests were used for the other comparisons. HPF, high power field; IQR, interquartile range; M2:M1, M2 and M1 macrophage ratio; MVD, microvessel density; PitNET, pituitary neuroendocrine tumour; TMVA, total microvessel area.
