# Supplementary material for: The role of the tumour microenvironment in the angiogenesis of pituitary tumours
Source: Endocrine. 2020 Sep 18;70(3):593–606. doi: 10.1007/s12020-020-02478-z (PMC7674353; doi:10.1007/s12020-020-02478-z)
Supplement: Supplementary file 4 — Supplemental Table 4 [file 12020_2020_2478_MOESM4_ESM.docx]

|  | **MVD** | **TMVA** | **Perimeter** | **Feret’s diameter** | **Area per vessel** | **Roundness** |
| --- | --- | --- | --- | --- | --- | --- |
| **PitNET-infiltrating macrophages** *[Median (IQR)]*  < 6% (n=11)  ≥ 6% (n=5) | 21.0 (17.3-36.7)  40.7 (33.8-71.3)  *p*=0.152 | 6.3 (4.9-9.9)  8.8 (5.8-14.4)  *p*=0.461 | 110.9 (97.9-132.9)  97.9 (87.4-110.8)  *p*=0.162 | 46.4 (40.6-52.1)  40.2 (35.7-43.9)  *p*=0.149 | 0.25 (0.19-0.41)  0.18 (0.16-0.22)  *p*=0.215 | 0.44 (0.41-0.51)  0.48 (0.45-0.49)  *p*=0.464 |
| **PitNET-infiltrating CD8+ T cells** *[Median (IQR)]*  < 1% (n=5)  ≥ 1% (n=11) | 32.3 (17.5-48.2)  32.3 (20.3-40.7)  *p*=0.644 | 8.1 (5.3-8.7)  6.0 (5.4-14.4)  *p*=0.444 | 108.8 (93.5-137.1)  109.9 (97.5-132.1)  *p*=0.960 | 45.5 (38.5-57.3)  44.7 (39.6-50.7)  *p*=0.868 | 0.22 (0.18-0.33)  0.22 (0.16-0.31)  *p*=0.678 | 0.44 (0.38-0.49)  0.48 (0.43-0.51)  *p*=0.189 |
| **PitNET-infiltrating CD4+ T cells** *[Median (IQR)]*  < 1% (n=9)  ≥ 1% (n=7) | 20.3 (16.3-34.5)  40.7 (32.3-87.0)  ***p*=0.044** | 5.9 (4.8-8.5)  8.9 (6.0-15.9)  ***p*=0.032** | 109.9 (98.2-147.7)  97.9 (97.5-119.2)  *p*=0.259 | 46.4 (39.7-59.4)  40.6 (39.6-47.5)  *p*=0.200 | 0.22 (0.17-0.36)  0.22 (0.15-0.29)  *p*=0.477 | 0.43 (0.41-0.47)  0.49 (0.48-0.53)  ***p*=0.004** |
| **PitNET-infiltrating B cells** *[Median (IQR)]*  < 0.5% (n=5)  ≥ 0.5% (n=11) | 19.7 (13.0-43.3)  36.7 (21.0-40.7)  *p*=0.251 | 6.3 (4.9-9.4)  8.1 (5.4-14.4)  *p*=0.367 | 110.9 (98.2-178.3)  108.8 (97.5-119.2)  *p*=0.254 | 46.4 (39.7-73.8)  44.7 (39.6-47.5)  *p*=0.226 | 0.22 (0.17-0.67)  0.22 (0.16-0.29)  *p*=0.381 | 0.43 (0.38-0.46)  0.48 (0.44-0.51)  ***p*=0.048** |
| **PitNET-infiltrating neutrophils** *[Median (IQR)]*  < 0.5% (n=6)  ≥ 0.5% (n=10) | 34.5 (20.2-63.5)  31.7 (16.8-40.7)  *p*=0.556 | 8.5 (4.2-16.9)  6.2 (5.6-9.1)  *p*=0.394 | 110.4 (103.8-118.9)  102.7 (94.6-139.9)  *p*=0.718 | 45.9 (42.6-47.8)  41.8 (38.5-55.0)  *p*=0.751 | 0.22 (0.18-0.29)  0.21 (0.16-0.33)  *p*=0.602 | 0.46 (0.42-0.49)  0.48 (0.42-0.52)  *p*=0.767 |
| **PitNET-infiltrating FOXP3+ T cells** *[Median (IQR)]*  < 0.3% (n=9)  ≥ 0.3% (n=7) | 36.7 (26.0-71.3)  20.3 (15.3-36.7)  *p*=0.071 | 8.1 (5.8-11.6)  6.3 (4.9-9.9)  *p*=0.617 | 107.3 (87.4-112.5)  132.1 (97.9-163.4)  ***p*=0.029** | 43.1 (35.7-45.9)  50.7 (40.2-68.1)  ***p*=0.033** | 0.21 (0.16-0.24)  0.31 (0.19-0.43)  *p*=0.137 | 0.48 (0.44-0.51)  0.43 (0.40-0.49)  *p*=0.195 |
| **Immune cell ratios** *[Spearman’s correlation rho (p)]*  M2:M1  CD8:CD4  CD8:FOXP3  CD68:FOXP3 | 0.162 (*p*=0.548)  -0.284 (*p*=0.286)  0.392 (*p*=0.134)  0.377 (*p*=0.150) | 0.494 (*p*=0.052)  -0.147 (*p*=0.587)  0.109 (*p*=0.688)  0.074 (*p*=0.787) | 0.208 (*p*=0.440)  0.094 (*p*=0.729)  -0.414 (*p*=0.111)  -0.372 (*p*=0.156) | 0.117 (*p*=0.666)  0.143 (*p*=0.598)  -0.399 (*p*=0.126)  -0.386 (*p*=0.140) | 0.149 (*p*=0.583)  0.124 (*p*=0.649)  -0.380 (*p*=0.147)  -0.332 (*p*=0.208) | 0.205 (*p*=0.446)  -0.097 (*p*=0.720)  0.541 (***p*=0.030**)  0.292 (*p*=0.272) |

**Supplemental Table 4: Correlation between infiltrating immune cells and angiogenesis in NF-PitNETs**

PitNET-infiltrating immune cell and angiogenesis data are shown for the cohort of NF-PitNETs (n=16). Microvessel density (MVD) is expressed in vessels/HPF; total microvessel area (TMVA) is expressed in % of the HPF; perimeter and Feret’s diameter are expressed in µm; area per vessel is expressed in % of the HPF; vessel roundness correspond to a value comprised between 0 and 1 (1=perfect circle). The PitNET-infiltrating immune cell thresholds considered here were the same as those previously published in [9]. The correlations between immune cell ratios and vessel parameters were determined by the Spearman’s correlation coefficient rho. Mann Whitney U tests were used for the other comparisons. HPF, high power field; IQR, interquartile range; M2:M1, M2 and M1 macrophage ratio; NF-PitNET, non-functioning pituitary neuroendocrine tumour; MVD, microvessel density; PitNET, pituitary neuroendocrine tumour; TMVA, total microvessel area.
