# Supplementary material for: The role of the tumour microenvironment in the angiogenesis of pituitary tumours
Source: Endocrine. 2020 Sep 18;70(3):593–606. doi: 10.1007/s12020-020-02478-z (PMC7674353; doi:10.1007/s12020-020-02478-z)
Supplement: Supplementary file 5 — Supplemental Table 5 [file 12020_2020_2478_MOESM5_ESM.docx]

|  | **MVD** | **TMVA** | **Perimeter** | **Feret’s diameter** | **Area per vessel** | **Roundness** |
| --- | --- | --- | --- | --- | --- | --- |
| **PitNET-infiltrating macrophages** *[Median (IQR)]*  < 6% (n=6)  ≥ 6% (n=2) | 31.3 (27.7-44.3)  37.0 (19.0-.)  *p*=0.992 | 4.0 (3.4-6.1)  7.5 (5.6-.)  *p*=0.229 | 77.4 (68.8-89.9)  101.4 (99.5-.)  ***p*=0.007** | 31.9 (26.7-36.9)  41.1 (39.5-.)  ***p*=0.019** | 0.13 (0.11-0.16)  0.23 (0.17-.)  ***p*=0.031** | 0.48 (0.46-0.54)  0.47 (0.46-.)  *p*=0.441 |
| **PitNET-infiltrating CD8+ T cells** *[Median (IQR)]*  < 1% (n=1)  ≥ 1% (n=7) | 21.7  32.7 (29.7-55.0)  *p*=0.404 | 3.8  5.0 (3.5-9.4)  *p*=0.517 | 98.5  79.3 (70.9-99.5)  *p*=0.358 | 38.5  32.6 (27.2-39.5)  *p*=0.493 | 0.18  0.13 (0.12-0.17)  *p*=0.778 | 0.45  0.47 (0.46-0.53)  *p*=0.358 |
| **PitNET-infiltrating CD4+ T cells** *[Median (IQR)]*  < 1% (n=6)  ≥ 1% (n=2) | 31.3 (27.7-59.3)  27.0 (19.0-.)  *p*=0.408 | 4.4 (3.4-9.4)  4.9 (4.2-.)  *p*=0.723 | 81.3 (68.8-98.7)  91.3 (79.3-.)  *p*=0.502 | 33.8 (26.7-38.8)  37.6 (32.6-.)  *p*=0.400 | 0.14 (0.11-0.17)  0.21 (0.12-.)  *p*=0.210 | 0.47 (0.46-0.54)  0.47 (.-.)  *p*=0.330 |
| **PitNET-infiltrating B cells** *[Median (IQR)]*  < 0.5% (n=3)  ≥ 0.5% (n=5) | 32.7 (21.7-.)  30.0 (24.3-53.5)  *p*=0.963 | 5.0 (3.8-.)  4.2 (3.3-7.5)  *p*=0.666 | 98.5 (87.1-.)  75.4 (66.7-91.3)  *p*=0.132 | 38.5 (36.4-.)  31.3 (26.3-37.6)  *p*=0.173 | 0.17 (0.15-.)  0.12 (0.11-0.21)  *p*=0.793 | 0.46 (0.45-.)  0.48 (0.47-0.54)  *p*=0.060 |
| **PitNET-infiltrating neutrophils** *[Median (IQR)]*  < 0.5% (n=7)  ≥ 0.5% (n=1) | 32.7 (21.7-55.0)  30.0  *p*=0.713 | 5.0 (3.8-9.4)  3.2  *p*=0.372 | 87.1 (75.4-99.5)  70.9  *p*=0.365 | 36.4 (31.3-39.5)  27.2  *p*=0.244 | 0.15 (0.12-0.18)  0.11  *p*=0.405 | 0.47 (0.46-0.48)  0.53  *p*=0.191 |
| **PitNET-infiltrating FOXP3+ T cells** *[Median (IQR)]*  < 0.3% (n=3)  ≥ 0.3% (n=5) | 55.0 (21.7-.)  30.0 (24.3-33.8)  *p*=0.302 | 9.4 (3.8-.)  4.2 (3.3-5.3)  *p*=0.218 | 98.5 (75.4-.)  79.3 (66.7-95.2)  *p*=0.376 | 38.5 (31.3-.)  32.5 (26.3-39.5)  *p*=0.462 | 0.17 (0.13-.)  0.12 (0.11-0.22)  *p*=0.983 | 0.46 (0.45-.)  0.47 (0.47-0.54)  *p*=0.164 |
| **Immune cell ratios** *[Spearman’s correlation rho (p)]*  M2:M1  CD8:CD4  CD8:FOXP3  CD68:FOXP3 | 0.405 (*p*=0.320)  0.476 (*p*=0.233)  0.143 (*p*=0.736)  -0.238 (*p*=0.570) | 0.167 (*p*=0.693)  0.429 (*p*=0.289)  0.000 (*p*=1.000)  0.143 (*p*=0.736) | -0.500 (*p*=0.207)  0.048 (*p*=0.911)  -0.286 (*p*=0.493)  0.143 (*p*=0.736) | -0.500 (*p*=0.207)  0.048 (*p*=0.911)  -0.286 (*p*=0.493)  0.143 (*p*=0.736) | -0.333 (*p*=0.420)  0.119 (*p*=0.779)  0.000 (*p*=1.000)  0.405 (*p*=0.320) | 0.313 (*p*=0.450)  -0.241 (*p*=0.565)  -0.169 (*p*=0.690)  -0.313 (*p*=0.450) |

**Supplemental Table 5: Correlation between infiltrating immune cells and angiogenesis in somatotrophinomas**

PitNET-infiltrating immune cell and angiogenesis data are shown for the cohort of somatotrophinomas (n=8). Microvessel density (MVD) is expressed in vessels/HPF; total microvessel area (TMVA) is expressed in % of the HPF; perimeter and Feret’s diameter are expressed in µm; area per vessel is expressed in % of the HPF; vessel roundness correspond to a value comprised between 0 and 1 (1=perfect circle). The PitNET-infiltrating immune cell thresholds considered here were the same as those previously published in [9]. The correlations between immune cell ratios and vessel parameters were determined by the Spearman’s correlation coefficient rho. Mann Whitney U tests were used for the other comparisons. HPF, high power field; IQR, interquartile range; M2:M1, M2 and M1 macrophage ratio; MVD, microvessel density; PitNET, pituitary neuroendocrine tumour; TMVA, total microvessel area.
